# Agrometeorological and Agronomic Characterization of *Megathyrsus* Grasses Cultivated in Tropical Humid and Semi-Arid Conditions: A Multivariate Approach

**DOI:** 10.3389/fpls.2022.809377

**Published:** 2022-02-25

**Authors:** Vitor Hugo Maués Macedo, Nauara Moura Lage Filho, Antônio Marcos Quadros Cunha, Marcos Neves Lopes, Rodrigo Gregório da Silva, José Antônio Alves Cutrim Junior, Cristian Faturi, Magno José Duarte Cândido, Aníbal Coutinho do Rêgo

**Affiliations:** ^1^Institute of Health and Animal Production, Federal Rural University of Amazon, Belém, Brazil; ^2^Nucleus of Agricultural Sciences and Rural Development, Federal University of Pará, Castanhal, Brazil; ^3^Federal Institute of Education, Science and Technology of Piauí, Valença, Brazil; ^4^Federal Institute of Education, Science and Technology of Ceará, Limoeiro do Norte, Brazil; ^5^Federal Institute of Education, Science and Technology of Maranhão, Maracanã, Brazil; ^6^Department of Animal Science, Federal University of Ceará, Fortaleza, Brazil

**Keywords:** agrometeorology, growing conditions, *Megathyrsus*, multivariate analysis, pasture ecosystems, semi-arid, tropical humid

## Abstract

Variability in climatic conditions of low-latitude tropical grass cultivation can affect forage production dynamics. Pasture ecosystems are complex and preferably studied from a multifactorial point of view through multivariate approaches. Therefore, in this study, we characterized different growing conditions for grasses of the *Megathyrsus* genus through studies conducted in tropical humid and semi-arid conditions. We applied principal component, canonical correlation, and discriminant function analyses to the measurements of agronomic and agrometeorological variables in six studies with Guinea and Massai grasses. The principal component analysis, through the climatic characterization by the first principal component, reflects the contrast between water availability and nitrogen variables and energy supply. Agronomic characterization occurred through the distinction between the density of tillers, forage accumulation, and increase in height, versus the accumulation of stems and dead material. The canonical correlation analysis generated a correlation coefficient of 0.84 between the agronomic and agrometeorological variables. There was a contrast between the dead material accumulation and the other agronomic variables, while the agrometeorological variables showed characteristics similar to the first principal component. Discriminant function 1, with 70.36% separation power, distinguished the cultivation conditions based on the study locations. Grass cultivars were differentiated by discriminant function 2, with a 19.20% separation power. From a multivariate variability analysis, despite the similarities of radiation and temperature in the regions studied, the availability of water and nutrients and measurements of agronomic variables can aid in future modeling studies on forage production.

## Introduction

Pasture ecosystems at low latitudes (<10°) show little variation in photoperiod and temperature but exhibit important differences in other climatic factors, such as rainfall. In these areas, depending on the climate in which they are located, tropical forages have limited growth from a climatic point of view, usually due to water availability (Santos et al., 2013). Therefore, close to the equator, the water regime dictates plant growth both in a humid tropical climate and a semi-arid climate. Such weather types are present at lower latitudes on four of the seven continents. Humid tropical climates are observed in South American countries, such as those of the Amazon region, as well as in the central region of the Congo in Africa, and the Indonesian islands of Asia, all of which have humid tropical forests constituting the main biome. A semi-arid climate can be observed in northeastern Brazil, some African countries such as Ethiopia, Kenya, Somalia, and Tanzânia, and in northern Australia (Peel et al., 2007).

Forage plant growth and consequently, canopy productivity are the result of the genotype and its related environment (Durand et al., 1991; Simeão et al., 2021). Environmental factors refer to the edaphoclimatic conditions of plant cultivation, including aspects related to soil (texture, density, and fertility) and climate (temperature, humidity, and photoperiod). The water demand of plants depends mainly on their metabolic requirements, which are linked to characteristics such as stomatal conductance, transpiration rate, and leaf area. These characteristics vary according to the stage of development (Tardieu, 2013). Water demand is also determined by factors such as leaf surface evapotranspiration, which is dependent on radiation, temperature, air humidity, wind speed, and leaf surface properties (Rind et al., 1990). In tropical pastures, evapotranspiration, mean temperature, and solar radiation influence total forage accumulation, leaf accumulation, tiller population density, and nutritional value (Lage Filho et al., 2021; Macedo et al., 2021; Tapia et al., 2021). As for aspects related to the soil, nitrogen (N) is the most important nutrient in tissue flow, and its assimilation may be limited by a water deficit (Onillon et al., 1995). Therefore, when climate conditions are favorable and nitrogen supply is adequate, studies in the literature support that the growth of tropical grasses, especially of the genus *Megathyrsus*, will be rapid, as there will be an increase in regrowth vigor and a reduction in the interval between grazing (Oliveira et al., 2020).

Cultivated tropical pasture ecosystems represent the main food source for many herds worldwide (Silva et al., 2013). Understanding the relationship between the agronomic characteristics of the grasses that make up such systems, and the agrometeorological conditions of these regions would increase the knowledge of the interactions between these factors in pasture ecosystems. Therefore, multivariate analyses of the factors related to climatic influences exerted on plants and the dynamics of growth and biomass production can provide important information through a systemic view of the ecosystem (Yeater et al., 2014; Araújo Júnior et al., 2021). In addition, the exploration of data using multivariate analysis can contribute to research with direct modeling applied to plant growth (Qiu et al., 2016), although little research has been previously conducted on the prediction of forage accumulation in tropical conditions.

Regions located close to the equator, such as humid and semi-arid tropical regions, may have climatic factors that can distinguish them, such as variables related to humidity and the water regime (Alvares et al., 2013). Production systems in these drier places use technologies such as irrigation (Araújo Júnior et al., 2021), which is often not required in humid tropical regions. Thus, agrometeorological characterization and analysis of agronomic variables of forage plants grown in different regions can provide valuable information on how different growing conditions interact with their environment, and how these conditions can be distinguished. Conducting trials under different climates helps in understanding the climatic influences on the growth and development of forage crops, and contributes to modeling studies involving climate action in tropical forage grasses that are highly responsive to change, such as those of the genus *Megathyrsus*.

This study aimed to understand the growth dynamics of grasses of the genus *Megathyrsus* under different growing conditions in humid and semi-arid tropical regions, and to answer the following questions: How are the different growing conditions characterized in relation to the indices that group agronomic and agrometeorological variables? How do agrometeorological variables relate to the agronomic variables measured under these growing conditions?, and How can discriminant functions be described that can distinguish cultivation conditions regarding forage species evaluated in the humid and semi-arid tropical regions of Brazil?

## Materials and Methods

### Experimental Sites

**Figure 1:**
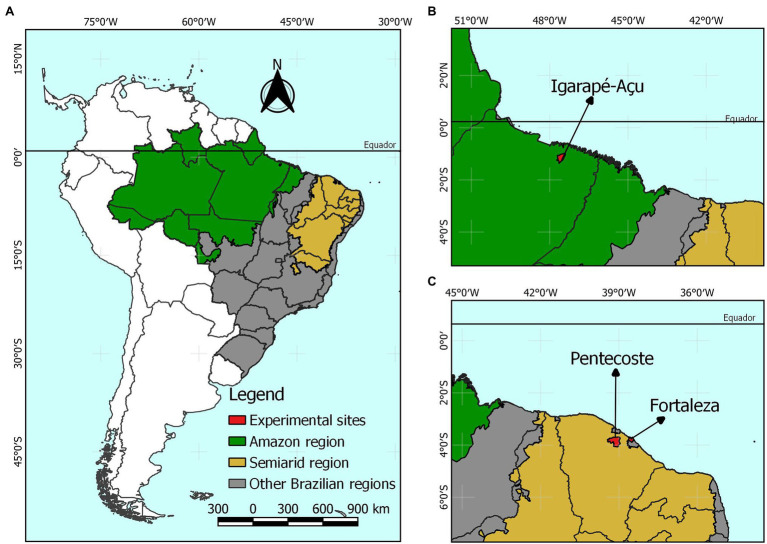
Location map of experimental sites. **(A)** Representation of the delimitation of the humid tropical region of the Amazon and the semi-arid region in Brazil. **(B)** Location of the Igarapé-Açu experimental site. **(C)** Location of the experimental sites of Fortaleza and Pentecoste.

Data were retrieved from four experimental trials with Guinea grass (*Megathyrsus maximus* (syn. *Panicum maximum*; Jacq.) B.K. Simon & S.W.L. Jacobs “Guinea”). Two trials were conducted in the municipality of Igarapé-Açu (01°07′ S, 47°36′ W, 47 m altitude), state of Pará, and two in Pentecoste (03°48′ S, 49°19′ W, 71 m altitude), state of Ceará. Two other experiments with Massai grass (*Megathyrsus maximus* × *Megathyrsus infestus* (Peters) B.K. Simon & S.W.L. Jacobs “Massai”) were performed in Igarapé-Açu and Fortaleza (03°44′ S, 38°34′ W, 20 m altitude) in the states of Pará and Ceará, respectively. The town of Igarapé-Açu is located in the eastern region of the Amazon biome, has a rainy climate with a short dry season, and is classified as type Am using the Köppen classification (Alvares et al., 2013) tropical humid monsoon. The Pentecoste experimental field is located in the Brazilian semi-arid region, with climate type “BSwh” according to the Köppen classification, indicating a dry climate with a short wet season. Fortaleza is a coastal city in northeastern Brazil located near the Brazilian semi-arid region. According to the Köppen classification, Fortaleza has a tropical savanna climate of the Aw’ type with dry-winter characteristics. (Figure 1). The experiments were named GG.IGA.15 (Guinea grass in Igarapé-Açu during the year 2015), GG.IGA.17-18 (Guinea grass in Igarapé-Açu during 2017 and 2018), GG.PEN.03 (Guinea grass in Pentecost during the year 2003), GG.PEN.05-06 (Guinea grass in Pentecost during 2005 and 2006), MG.IGA.15 (Massai grass in Igarapé-Açu during the year 2015), and MG.FOR.09 (Massai grass in Fortaleza during the year 2009). The agrometeorological characteristics and the details of the growing conditions of grasses of the genus *Megathyrsus* are presented in Table 1.

**Table 1:** Means and standard deviations (SD) of agrometeorological and cultivation conditions during data collection for experimental trials.

ID^a^	Grass	Place	Weather condition	Soil^b^	Photo^c^	SR^d^	Tmean^e^	Eto^f^	Prec^g^	WA^h^	AWC^i^	ETa^j^
GG.IGA.15	Guinea Grass	Igarapé-Açu	Humid	Yellow Oxisol	12.00 ± 0.02	461.93 ± 76.08	27.21 ± 1.33	8.40 ± 1.37	4.95 ± 8.86	4.95 ± 8.86	100	3.78 ± 3.45
GG.IGA.17-18	Guinea Grass	Igarapé-Açu	Humid	Yellow Oxisol	12.00 ± 0.02	443.57 ± 90.32	26.56 ± 1.24	8.01 ± 1.59	6.40 ± 13.88	6.40 ± 13.88	100	4.26 ± 3.14
GG.PEN.03	Guinea Grass	Pentecoste	Semi-Arid	Fluvisol	12.00 ± 0.05	215.26 ± 25.60	26.78 ± 0.79	4.51 ± 0.53	0.04 ± 0.40	12.60 ± 20.46	56	4.18 ± 0.57
GG.PEN.05-06	Guinea Grass	Pentecoste	Semi-Arid	Fluvisol	12.08 ± 0.02	200.53 ± 34.04	30.30 ± 1.11	5.15 ± 0.98	0.93 ± 4.08	12.50 ± 20.80	56	4.69 ± 0.89
MG.IGA.15	Massai Grass	Igarapé-Açu	Humid	Yellow Oxisol	11.98 ± 0.01	433.61 ± 89.16	26.00 ± 0.85	7.79 ± 1.56	10.02 ± 11.76	10.02 ± 11.76	100	6.81 ± 1.53
MG.FOR.09	Massai Grass	Fortaleza	Semi-Arid	Yellow Ultisol	11.97 ± 0.04	284.60 ± 40.39	26.76 ± 0.82	5.82 ± 0.89	1.89 ± 7.26	8.82 ± 12.56	32	5.22 ± 0.88

a *Identification of the experimental trial*.

b *Soil classification according to IUSS Working Group WRB (2015)*.

c *Average photoperiod (hours day^−1^)*.

d *Average of daily solar radiation (W m^−2^)*.

e *Mean daily temperature (°C)*.

f *Average of reference evapotranspiration (mm day^−1^)*.

g *Precipitation (rainfall; mm day^−1^).*

h *Water applied from rain and/or irrigation (mm day^−1^)*.

i *Available water capacity (mm)*.

j *Average of actual evapotranspiration (mm)*.

### Description of Experimental Trials and Growing Conditions

#### Experiments With Mechanized Forage Harvesting Without the Use of Irrigation

The GG.IGA.15 experimental trial was conducted at the Experimental Farm of Igarapé-Açu (FEIGA) of the Federal Rural University of Amazônia (UFRA). The grass was sown by hand on March 6, 2014, with a sowing rate equivalent to 40 pure seeds m^−2^ after tillage. In this trial, treatments with different harvest frequencies were tested in Guinea grass based on fixed days (14, 21, 28, 35, 42, and 49 days) of the rest period. Thus, the cuts and collection of biomass were carried out after each period of days established by the treatments. The forage was harvested using a hedge trimmer. The area was divided into 30 plots of 12 m^2^ (3 m × 4 m), with corridors spaced 1 m apart. The experimental design was a randomized block with five replicates per treatment. We used data collected between March 14, 2015, and January 2, 2016, which covered collections in both the rainy and dry seasons of 2015. Irrigation was not used in this trial; therefore, during the rainy season, nitrogen fertilizers were applied at a rate of 200 kg N ha^−1^ year^−1^ in the form of urea (45% N). The plots with 14, 21, 28, 35, 42, and 49 days of the rest period received doses equivalent to 17, 25, 34, 42, 51, and 59 kg N ha^−1^ cycle^−1^, respectively. For more details on this study, see Macedo et al. (2021).

The GG.IGA.17-18 test used the same experimental units as the GG.IGA.15 test. This study evaluated the effect of different defoliation intensities based on the residue height (15, 25, 35, 45, and 55 cm) when the Guinea grass canopy reached 95% light interception, measured using an AccuPAR LP-80 canopy analyzer (Decagon^®^). The experimental design was a randomized block with six replicates per treatment. The data from this study were collected from September 2, 2017 to September 12, 2018 and included collections during the dry and rainy seasons. For more details about this study, see Lage Filho et al. (2021). In both GG.IGA.15 and GG.IGA.17-18, nitrogen fertilizers were applied during the rainy season at a rate of 200 kg N ha^−1^ year^−1^ in the form of urea (45% N), and defoliations were performed using mechanical cutting.

In the experimental trial MG.IGA.15, the grass was sown by hand on May 20, 2014, with a sowing rate equivalent to 45 pure seeds m^−2^ after tillage. The Massai grass was subjected to six treatments: five doses of nitrogen fertilization (100, 200, 300, 400, and 500 kg ha^−1^ year^−1^), in six fixed applications throughout the experimental period, and a control treatment (no nitrogen), with five replications in a completely randomized design. The area was divided into 30 plots of 12 m^2^ (3 m × 4 m), with corridors spaced 1 m apart. Defoliation was performed using mechanized cutting when light interception reached 95%, as measured using an AccuPAR LP-80 canopy analyzer (Decagon^®^). The experiment was conducted at FEIGA from February 14, 2015, to August 5, 2015. For more details on this study, refer to Cunha et al. (in press).

#### Sheep Grazing Experiments Using Irrigation

The experimental trial GG.PEN.03 was conducted in the advanced Teaching and Research Unit in Forage (NEEF) in Pentecoste-CE. The grass was sown manually in January 2003. Guinea grass was subjected to three rest periods, defined as a function of the time needed for expansion of 1.5, 2.5, and 3.5 new sheets per tiller, with two repetitions per treatment. Therefore, the area was divided into six rotating stocking systems, two for each rest period, for evaluation. The data from this study included collections from August 3, 2003, to November 8, 2003 and covered the dry period in the region. Harvesting was performed by sheep in an area under sprinkler irrigation with a water depth of approximately 11.4 mm day^−1^ and a four-day watering shift. Nitrogen fertilizers were applied at a rate of 160 kg N ha^−1^ year^−1^ in the form of urea (45% N). The design was completely randomized. For more details on this study, see Silva et al. (2007).

The experimental trial GG.PEN.05–06 was performed at NEEF in Pentecoste-CE in the same area as the trial GG.PEN.03. Guinea grass was subjected to three rest periods based on the time required for the canopy to reach 85, 95, and 97% light interception, in combination with two post-grazing residues based on a residual leaf area index of 1.0 or 1.8 for a total of six treatments. Light interception and leaf area index were measured using an AccuPAR LP-80 canopy analyzer (Decagon^®^). The design was completely randomized with four replicates, totaling 24 experimental units divided into 24 paddocks. The data from this study were collected from October 25, 2005, to March 7, 2006. Harvesting was performed by sheep in an area under fixed sprinkler irrigation with a water depth of approximately 11.4 mm day^−1^ and a four-day watering shift. Nitrogen fertilizers were applied at a rate of 220 kg N ha^−1^ year^−1^ in the form of urea (45% N). For more details about this study, see Cutrim Junior et al. (2011).

The experimental test MG.FOR.09 was performed in the experimental field of NEEF in Fortaleza, CE. The grass was sown by hand in September 2008, with a sowing rate equivalent to 2 kg of pure seeds ha^−1^ after tillage. Massai grass was subjected to increasing doses of nitrogen (0-control; 400, 800, and 1,200 kg ha^−1^ year^−1^) under a fixed sprinkler irrigated area with a liquid depth of 7.0 mm day^−1^ and watering shift of 3 days, in an intermittent stocking system grazed by sheep. The experimental design was completely randomized with two repetitions and evaluations at each regrowth cycle lasting 22, 18, 16, and 13 days for the control treatments (without fertilization), and 400, 800, and 1,200 kg of nitrogen fertilization, respectively. The experiment was conducted at FEIGA from July 14, 2009, to October 18, 2009. For more details on this study, see Lopes et al. (2016).

A summary of the experimental and management conditions of each trial is presented in Table 2.

**Table 2:** Characterization of the experimental and management conditions of each experimental trial.

ID^a^	Planting date	Trial period	Experimental design	Treatments	Nitrogen fertilization	Type of harvest	Use of irrigation
GG.IGA.15	March 6, 2014	March 14, 2015 to January 2, 2016	Randomized block design	Harvest frequencies based on fixed days (14, 21, 28, 35, 42, and 49 days) of the rest period	200 kg of N ha^−1^ year^−1^ in the form of urea (45% N)	Mechanical cutting	No
GG.IGA.17-18	March 6, 2014	September 2, 2017 to September 12, 2018	Randomized block design	Harvest intensities based on the residue height (15, 25, 35, 45, and 55 cm)	200 kg of N ha^−1^ year^−1^ in the form of urea (45% N)	Mechanical cutting	No
GG.PEN.03	January 2003	August 3, 2003 to November 8, 2003	Completely randomized design	Harvest frequencies based on time needed for expansion of 1.5, 2.5, and 3.5 new sheets per tiller	160 kg of N ha^−1^ year^−1^ in the form of urea (45% N)	Grazed by sheep	Yes
GG.PEN.05–06	January 2003	October 25, 2005 to March 7, 2006	Completely randomized design	Harvest frequencies in combination with post-grazing residues	220 kg of N ha^−1^ year^−1^ in the form of urea (45% N).	Grazed by sheep	Yes
MG.IGA.15	May 20, 2014	February 14, 2015 to August 5, 2015	Completely randomized design	Nitrogen fertilization (control, 100, 200, 300, 400, and 500 kg ha^−1^ year^−1^)	Same as treatments	Mechanical cutting	No
MG.FOR.09	September 2008	July 14, 2009 to October 18, 2009	Completely randomized design	Nitrogen fertilization (control, 400, 800, and 1,200 kg ha^−1^ year^−1^)	Same as treatments	Grazed by sheep	Yes

a *Identification of the experimental trial*.

### Measured Agronomic Variables

In all trials, the common variables measured prior to defoliation were biomass accumulation and its morphological components, tiller population density (TPD), and canopy height increment (CHI). The number of days of the rest period (RP) between pastures was also determined.

The accumulation of biomass was measured by destructive collections with the use of known area frames used in each experimental trial, which were converted to hectares, considering the forage above the height of a particular residue in each trial. In the GG.IGA.15, GG.IGA.17-18, and MG.IGA.15 trials, forage collection was performed using two samplings in a 0.5 m^2^ frame (1.0 m 0.5 m). In the GG.PEN.03 and GG.PEN.05–06 trials, collection was performed using two samplings in a 1.0 m^2^ frame (1.0 m × 1.0 m). In the MG.FOR.09 trial, the collection was performed using two samplings in a frame of 0.0625 m^2^ (0.25 m × 0.25 m).

From biomass collection, the total forage accumulation (FA) and the morphological composition were determined through the separation of its plant components, obtaining the leaf blade accumulation (LBA), stem accumulation (stem + sheath; SA), and dead material accumulation (DMA). Each sample was placed in a forced ventilation oven (55°C to constant weight) to determine biomass accumulation in terms of dry matter. The CHI was obtained by the difference in canopy height before and after defoliation, measured with the aid of a ruler graduated in centimeters (Barthram, 1986). The TPD was estimated by counting the live tillers within a known area frame and converting to the number of tillers per square meter.

### Agrometeorological Variables

The mean temperature (Tmean) and precipitation (Prec) data for the municipality of Igarapé-Açu were obtained through the conventional meteorological station of Brazilian Agricultural Research Corporation (Embrapa), 900 m from the experiments. The same meteorological data, along with air humidity and wind speed, were obtained by the UFC automatic meteorological station at 800 and 550 m from the experimental area in the municipalities of Pentecoste and Fortaleza, respectively. Due to the absence of radiation measurements at some stations, global solar radiation (SR) data for the three sites were retrieved from the National Solar Radiation Database (NSRDB).

The reference evapotranspiration (ETo) of the cities of Fortaleza and Pentecoste was provided by the Penman-Monteith FAO 56 (FAO, 1998), according to the following equation:


$$ETo=\frac{0.408\Delta \left(R_{n}-G\right)+\gamma \frac{900}{T_{\mathrm{mean}}+273}u_{2}\left(e_{s}-e_{a}\right)}{\Delta +\gamma \left(1+0.34u_{2}\right)}$$

where ∆ is the slope vapor pressure curve as a function of temperature (kPa°C^−1^), and Rn is the net radiation at the crop surface (MJ.m^−2^ day^−1^); G is the soil heat flux density (MJ m^−2^ day^−1^); γ is the psychometric constant (kPa °C^−1^); T_mean_ is the mean between maximum and minimum temperature (°C); u_2_ is the wind speed (m s^−1^); and (e_s_-e_a_) is the saturation vapor pressure deficit (kPa).

The absence of wind speed and humidity data at the Igarapé-Açu meteorological station required that the ETo be calculated according to the method of Turc (1961), which represents an adequate estimate of the ETo for the region (Silva Júnior et al., 2017; Farias et al., 2019):


$$ETo=\frac{0.013T_{\mathrm{mean}}}{T_{\mathrm{mean}}+15}\left(23.9R_{s}+50\right)$$

where T_mean_ is the mean between the maximum and minimum temperatures (°C) and R_s_ is the solar global radiation (MJ m^−2^ day^−1^).

The water index (WI) was obtained from the relationship between the actual evapotranspiration (ETa) and reference (ETo):


$$WI=\frac{\mathrm{actual}\;\mathrm{evapotranspiration}}{\mathrm{reference}\;\mathrm{evapotranspiration}}$$

ETa was calculated from the preparation of the sequential water balance on a daily scale according to Thornthwaite and Mather (1955), based on the available water capacity (AWC), which was different for each location (Table 1). The AWC of the soil was calculated as the difference between the field capacity (FC) and the permanent wilting point (PWP). FC and PWP were obtained from undeformed soil samples saturated with water and subjected to tension of 10 and 1,500 kPA, respectively, in a Richards chamber.

Water from irrigation and/or precipitation was the applied water variable (WA). The supplied nitrogen (SN) was the agrometeorological variable for plant nitrogen availability in the form of fertilization.

### Statistical Analysis

The mean and/or some of the values of the agrometeorological (Tmean, SR, ETo, ETa, WI, WA, and SN) and agronomic (TPD, CHI, RP, FA, LBA, SA, and DMA) variables related to the regrowth period of each forage production cycle were used to form two groups (the group of agrometeorological variables and the group of agronomic variables).

The characterization of the observations from each trial was verified using principal component analysis (PC) through the generation of indices summarizing the agrometeorological and agronomic variables and represented in biplot graphs. The eigenvalues and eigenvectors were calculated from the correlation matrix to ensure that the results were not biased by large numerical variables. The choice of the number of components was based on the PC that obtained eigenvalues greater than 1, according to the Kaiser criterion (Kaiser, 1958).

To analyze how agronomic and agrometeorological variables are related, the data were subjected to canonical correlation analysis, whereby the participation of each variable in the generation of canonical indices was determined by the correlation of the canonical and the original variable.

Agrometeorological and agronomic variables were used in a discriminant function analysis to verify the functions responsible for maximizing the difference between the trials, based on the grass cultivar and the location of the experiment. To determine which variables were responsible for the separation, the generated discriminant functions were correlated with the original variables, and the Pearson correlation coefficients were described in a biplot graph. To verify how the assays differed, the new variables (discriminating functions) generated were subjected to the F test for mean differences and Tukey’s test for mean comparisons, both at a significance level of 0.05. The studentized residues of the model were subjected to the identification of outliers (values above 3.0 and below −3.0) and normality using the Shapiro–Wilk test. The homoscedasticity of variances test was performed, and the tests that showed heterogeneous variances were grouped to the model using the restricted maximum likelihood method.

The vectors that describe the variables in the biplot graphs generated in the principal component and discriminant analyses were multiplied by factors 10 and 20, respectively. Such adjustments were made to improve the scaling of vectors in the graph. In all the analyses described, R software (R Core Team, 2019) was used as a tool for data processing.

## Results

### How Are the Different Cultivation Conditions Characterized in Relation to the Indices That Group Agronomic and Agrometeorological Variables?

**Figure 2:**
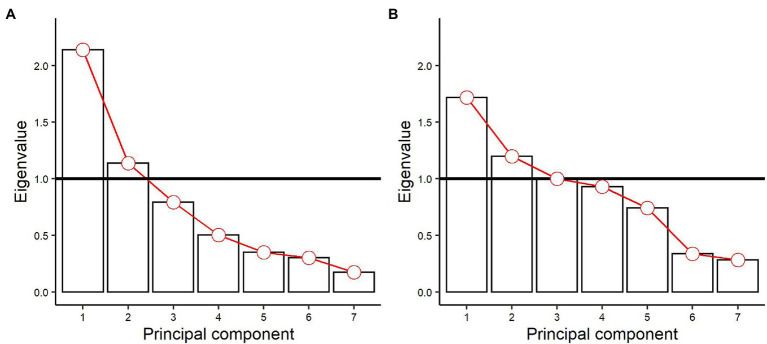
Variance explained by each principal component, through their respective eigenvalues. **(A)** Eigenvalues of the principal components referring to agrometeorological variables. **(B)** Eigenvalues of the principal components referring to agronomic variables.

Choosing the PC number using the Kaiser criterion allowed the selection of the first two components for characterization based on agrometeorological variables and the first three components based on agronomic variables, as observed in the scree plot graphs (Figure 2).

**Figure 3:**
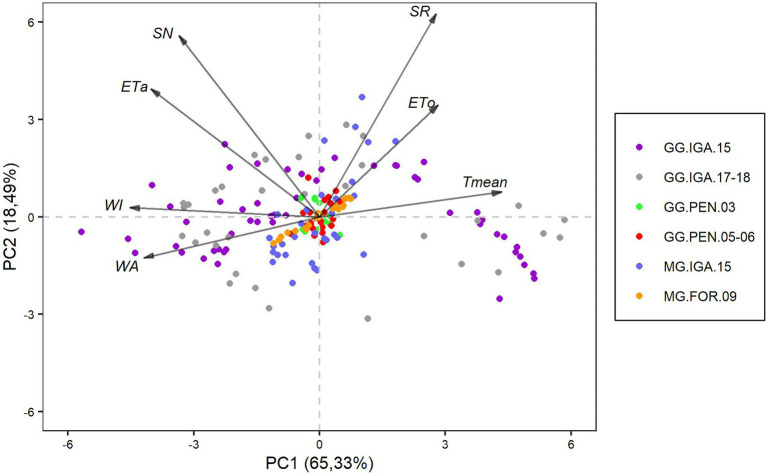
Biplot representation of the first two main components obtained from the agrometeorological variables, with observations from the six types of trials with cultivars of the species *Megathyrsus maximus*. ETa, Actual Evapotranspiration; ETo, Reference Evapotranspiration; SN, Supplied nitrogen; SR, Solar radiation; Tmean, Mean temperature; WA, Water applied; and WI, Water index.

The characterization of the six trials, through the agrometeorological variables, was influenced by the first main component (PC1; Figure 3), which accounted for 65.33% of the data variation. PC1 (Figure 3) demonstrated a contrast between variables related to water availability (ETa, WA, and WI) and SN versus variables related to energy supply (SR and Tmean) that result in potential evapotranspiration (ETo). There was a greater variability in the tests that used Guinea grass in the municipality of Igarapé-Açu (GG.IGA.15 and GG.IGA.17-18) than the other tests (Figure 3).

Principal component 2 (PC2, Figure 3) explained 18.49% of the data variation and was related to a greater variability of the humid tropical climate tests of the Amazon region, both for Massai grass and Guinea grass in relation to those in the Northeast region. With the exception of WA, the other agrometeorological variables showed positive coefficients for this component. There was also high participation for SR, SN, ETo, and ETa (Figure 3).

The biplot graph provides information related to the correlation between variables, where arrows in the same direction but opposite senses represent strong negative correlations between the variables. The ETo and Tmean showed strong negative correlations with WI and WA (Figure 3). In contrast, the arrows that show close directions, forming a small angle between them, are more positively correlated. This can be observed between the energy supply variables (SR, ETo, and Tmean) and the water availability variables (WI and WA) for ETa and SN.

**Figure 4:**
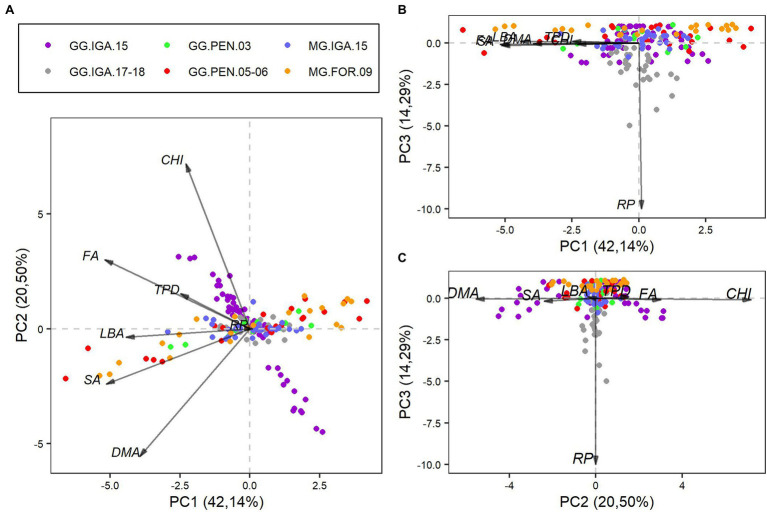
Biplot representation of the first three main components obtained from the agrometeorological variables, with observations from the six types of trials with cultivars of the species *Megathyrsus maximus*. **(A)** Bidirectional relationship of principal component 1 with principal component 2. **(B)** Bidirectional relationship of principal component 1 with principal component 3. **(C)** Bidirectional relationship of principal component 2 with principal component 3. CHI, Canopy height increment; DMA, Dead material accumulation; FA, Forage accumulation; LBA, Leaf blade accumulation; SA, Stem accumulation; and TPD, Tiller population density.

The first three main components of the agronomic variables accounted for 76.92% of the data variability. Principal component 1 (PC1, Figure 4), responsible for 42.14% of the variation, represents the growth of grasses, through an index that considers all agronomic variables, with low participation of RP. All trials were characterized by alterations in the high and low agronomic variable values (Figures 4A,B).

The second principal component (PC2, Figure 4), which held 20.50% of the data variability, was related to a contrast mainly between SA and DMA versus CHI, FA, and TPD, with a low participation of LBA. In this component, there was greater variability in the data from the Guinea grass trial in 2015 (Figures 4A,C). Principal component 3 (PC3, Figure 4) highlighted the importance of grass regrowth days. It accounted for 14.29% of the data variability and showed that this variation in the regrowth period between trials did not change substantially, except for some observations in the Guinea grass study in Igarapé-Açu during 2017 and 2018 (Figures 4B,C).

### How Do Agrometeorological Variables Relate to Agronomic Variables?

**Figure 5:**
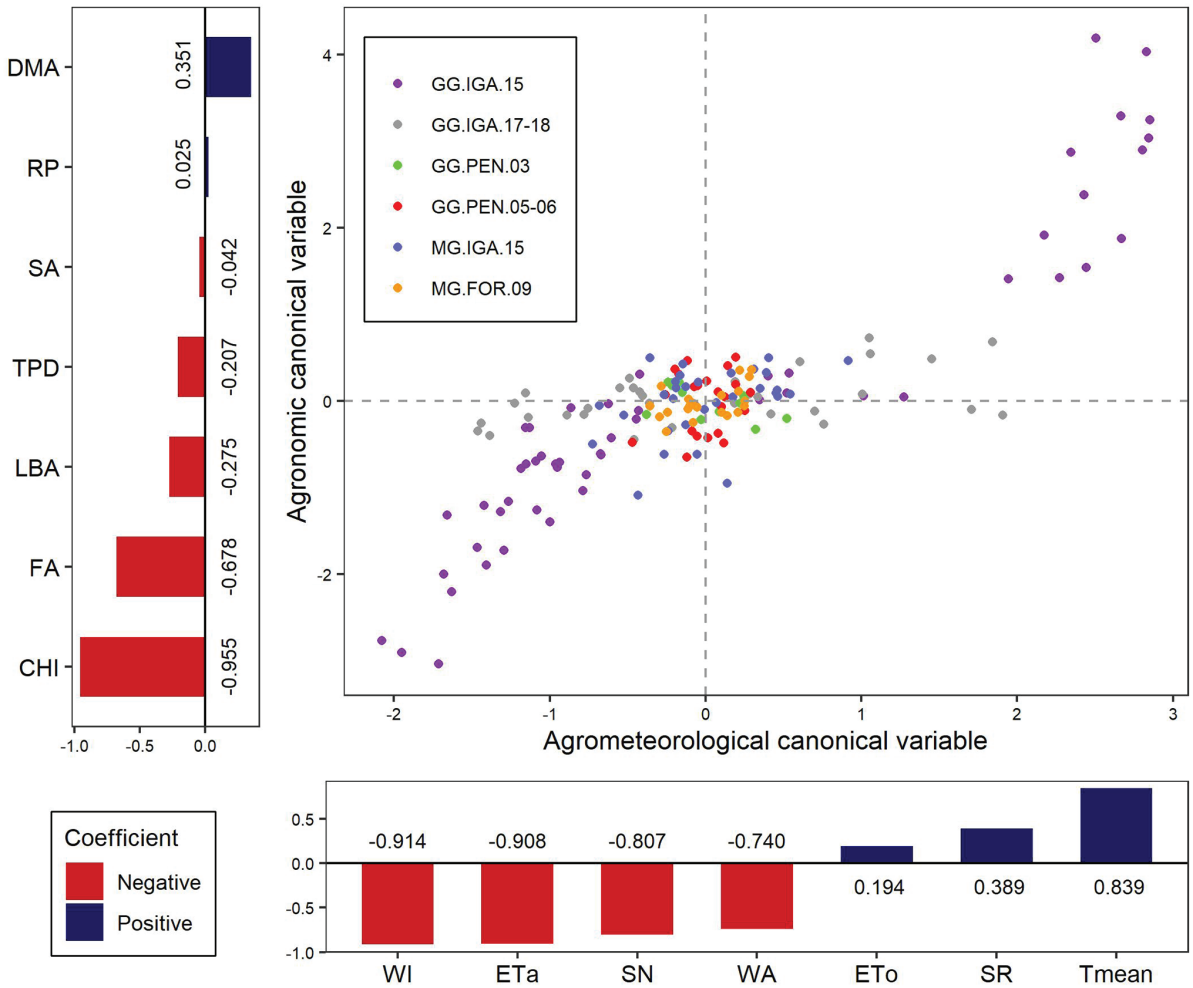
Graphic representation of the canonical correlation between the group of agrometeorological variables (Agrometeorological canonical variable) with the agronomical variables (Agronomic canonical variable). The bar graphs to the left and below the agronomic and agrometeorological variables, respectively, represent the influence of each original variable in the generation of the canonical variable through the value of their correlation coefficient. ETa, Actual evapotranspiration; ETo, Reference evapotranspiration; SN, Supplied nitrogen; SR, Solar radiation; Tmean, Mean temperature; WA, Water applied; WI, Water index; CHI, Canopy height increment; DMA, Dead material accumulation; FA, Forage accumulation; LBA, Leaf blade accumulation; SA, Stem accumulation; and TPD, Tiller population density.

The correlation between the canonical agrometeorological variables and their original counterparts showed a contrast between variables related to water availability (WA, ETa, and WI) with SN versus the variables related to energy supply in terms of temperature and radiation (ETo, SR, and Tmean; Figure 5). Through the correlation between the canonical agronomic variable and their original counterparts, it appears that former represented a contrast between DMA and the other variables (FA, LBA, TPD, and CHI), mainly with respect to the increase in height (Figure 5). SA and days of regrowth showed little participation in this canonical variable. Based on this relationship, environments with high Tmean, low water availability, and low nitrogen supply promote low plant growth, mainly in terms of lower FA and CHI, while accelerating the death of plant material characterized by high DMA.

The correlation coefficient between the agrometeorological and agronomic canonical variables was 0.84, which represents 76.94% of the variation explained by the first pair of canonical variables. The data from the trial with Guinea grass in Igarapé-Açu during 2015 were the most heterogeneous of all trials in terms of both the canonical agrometeorological and agronomic variables. The trial with Guinea grass in Igarapé-Açu between 2017 and 2018 showed some variability in the canonical agrometeorological variable, but with more homogeneous results for the canonical agronomic variable (Figure 5).

### How Can Discriminating Functions That Separate Experiments With Guinea and Massai Grasses Conducted in Distinct Regions, but at Similar Latitudes, Be Described?

**Figure 6:**
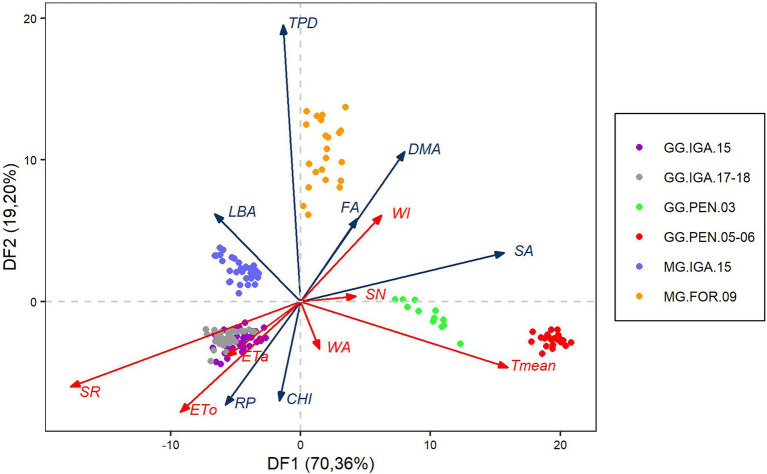
Biplot representation of the first two discriminant functions with observations from six types of trials with cultivars of the species *Megathyrsus maximus*. The red and blue arrows show the participation of each agrometeorological and agronomic variable, respectively, in the formation of the discriminant function. ETa, Actual evapotranspiration; ETo, Reference evapotranspiration; SN, Supplied nitrogen; SR, Solar radiation; Tmean, Mean temperature; WA, Water applied; WI, Water index; CHI, Canopy height increment; DMA, Dead material accumulation; FA, Forage accumulation; LBA, Leaf blade accumulation; SA, Stem accumulation; and TPD, Tiller population density.

The separation between the groups of experiments can be seen by the influence of two discriminant functions, which together represent 89.56% of the variation responsible for the maximum separation of the groups (Figure 6). Discriminant function 1 (DF1) revealed a contrast between the rest period and LBA versus FA, DMA, and SA. This discriminant function mainly separates the tests by region. The left side represents Igarapé-Açu, and the right denotes Fortaleza, and the extremes are the Pentecoste region. The data from semi-arid region were characterized by greater production of biomass and its components of the stalk and dead material, than those in the Amazon region, with emphasis on the tests with Guinea grass. For the agrometeorological variables, the separation of regions occurred through a contrast mainly between ETa, ETo, and SR on one side and between Tmean and WI on the other (Figure 6). DF1 had the highest mean for GG.PEN.05–06, followed by GG.PEN.03, and MG.FOR.09, for positive values on the axis. Regarding negative values, the MG.IGA.15 and GG.IGA.15 assays were equal, and the lowest values were found for the GG.IGA.17-18 study (Table 3).

**Table 3:** Difference between studies based on the comparison of the mean of the generated discriminant functions.

Studies	Discriminant functions	
	DF1^a^	DF2^b^
MG.IGA.15	−4.64 d	2.24 b
GG.IGA.15	−4.76 d	−2.82 d
GG.IGA.17-18	−5.67 e	−2.58 d
MG.FOR.09	1.78 c	10.50 a
GG.PEN.03	9.79 b	−0.96 c
GG.PEN.05-06	19.39 a	−2.69 d
value of *p*	<0.0001	<0.0001

a *Discriminant function 1*.

b *Discriminant function 2*.

*Means followed by the same letter in the column do not differ by Tukey’s test at 0.05*.

Discriminant function 2 (DF2) separated the grass cultivars. This function separates the tests that used Massai grass, characterized by higher TPD, biomass accumulation, and its morphological components, from the tests that evaluated Guinea grass, which had a greater increase in height and regrowth days. The agrometeorological variables were influenced by Tmean, SR, and ETo, in contrast to WI (Figure 6). DF2 had the highest mean for the MG.FOR.09 study, followed by MG.IGA.15 with the positive values on the axis. The GG.PEN.03 assay had the highest mean negative value, followed by the GG.IGA.15, GG.IGA.17-18, and GG.PEN.05–06 studies, which were equal (Table 3).

## Discussion

The relationship between agrometeorological and agronomic variables in tropical grasses has been little explored using multivariate approaches. A correct understanding of these interactions will help identify the most efficient use for tropical grass management according to region as will direct-to- research studies on grazing management and tropical grass growth modeling. The importance of this knowledge was confirmed in our study by the formation of indices that summarize the variable information into two groups based on agrometeorological variables, both by principal component and canonical correlation analysis. One group included variables related to energy supply in terms of radiation and temperature, and the other by water and nitrogen availability factors. The *Megathyrsus* sp. in different climatic conditions was distinguished mainly by the availability of water. This corroborates our hypothesis that even in regions located close to the equator, such as the Amazon and semi-arid regions of Brazil, there are climatic factors capable of discriminating them in terms of productive potential (Silva et al., 2019a).

PC1 (Figure 3) shows that the cultivation conditions with Guinea grass in Igarapé-Açu (GG.IGA.15 and GG.IGA.17-18) had greater data dispersion than the other trials. Climatic variability during the trials may explain the main cause of this difference. The tests in the semi-arid region involved a greater control of climatic conditions through water supply irrigation. In this region, radiation and temperature variability were not as pronounced; water supply through irrigation ensures continuous water availability, and there is no significant variability in actual evapotranspiration (Graham et al., 2016).

Similar to the town of the semi-arid region, Igarapé-Açu is located close to the equator (Figure 1). Consequently, the photoperiod, solar radiation, and temperature data did not vary substantially over the experimental period (Table 1). However, the variability observed in ETa, particularly in the GG.IGA.15 and GG.IGA.17-18 studies (Table 1), can be attributed to the cultivation conditions that included evaluations in both the rainy and the dry periods (Lage Filho et al., 2021; Macedo et al., 2021). In the MG.IGA.15 trial, the evaluations covered only the rainy season and the rainy-dry transition period.

PC1 also considered supply nitrogen, along with the water variables (Figure 3). The SN in Guinea grass trials in Igarapé-Açu showed greater variability due to the absence of nitrogen fertilization during the dry period, which was not required because of the lack of soil moisture (Kunrathm et al., 2018). The contrast observed by PC1 (Figure 3) was related to a greater dispersion of the variables related to water availability and SN than the energy supply and potential water loss variables, mainly in Igarapé-Açu. Based on this, we consider that statistical methods, such as principal component regression, can be potential alternatives for modeling studies with agrometeorological variables and soil properties, as observed in other studies, such as those by Zhou et al. (2021). These authors used principal component regression to relate climatic variables, soil properties, and plant characteristics to the spatial variability of the net exchange of CO_2_ between land and atmosphere, using data from croplands, pastures, and forests in different regions. Dispersion of observations in the Amazon region was observed in the second principal component (PC2, Figure 3), in which there was greater participation of the Massai grass trial (MG.IGA.15). In this trial, no evaluations were conducted during the dry season; therefore, the component was generated with most of the variables from only the positive side of the axis (Figure 3). For empirical modeling purposes, the variability in growing conditions of the GG.IGA.15 and GG.IGA.17-18 trials are important for obtaining models that can be generalized in prediction processes (Andrade et al., 2015).

We obtained three main components for the agronomic variables. All experimental tests showed some variability in the first principal component (PC1; Figures 4A,B). This is because the variation in observations is due to the treatments adopted in the trials, whether using different doses of nitrogen fertilization (trials with Massai grass) or different management techniques (trials with Guinea grass). In pasture production systems, it is common to adopt different management techniques based on grazing goals, which will depend on the technological level, production system, and possible edaphoclimatic variation in the region (Silva et al., 2019b,c; Macedo et al., 2021). These factors are indispensable when considering the changeability of response variables that are relevant for monitoring the structure and productivity of pastures.

Not only agrometeorological conditions are important in understanding and predicting productive variables in pastures. Other factors such as management of mechanistic models that estimate biomass production, such as CROPGRO (Bosi et al., 2020b; Brunetti et al., 2021) and APSIM (Bosi et al., 2020a; Gomes et al., 2020), are also relevant.

The second principal component (PC2; Figures 4A,C) mainly showed the larger variability in relation to the GG.IGA.15 trial, because the data from this trial reflected canopies with high and low values of agronomic variables due to use of rest days ranging from 14 to 49 (Macedo et al., 2021). The low participation of LBA in this study is linked to the small variability of this factor in the observed data. It is likely that the accumulation of leaf blades between canopies with different intervals between defoliations did not vary substantially because of TPD and the number of leaves per tiller. Canopies with higher RP had more leaves per tiller and wider and larger leaves, but lower TPD. Canopies with lower RP had fewer leaves per tiller and thinner leaves, but higher TPD. In general, compensation for the number of tillers did not modify the LBA (Sbrissia et al., 2010).

The third principal component (PC3; Figures 4B,C) highlighted the effect of regrowth days. Except for some data for the GG.IGA.17-18 trial, the trials presented a similar distribution of observations for this component. Fixed regrowth periods were not considered, and evaluations occurred during the dry period; therefore, the time for the canopy to reach 95% light interception (experiment goal) was high in some cycles of the GG.IGA.17-18 trial (Lage Filho et al., 2021). This may be related to the increased variability of observations in this trial for this component. Using management goals based on light interception in periods in which there are no favorable conditions for plant growth can be challenging, which has been described in other studies (Carnevalli et al., 2006).

The existing linear relationships between the set of agronomic and agrometeorological variables can be represented by canonical variables, whereby the maximum possible canonical correlation is desired (Araújo Júnior et al., 2021). Therefore, the high canonical correlation (0.84) between the agrometeorological and agronomic variables demonstrates the close interaction between these two groups. This influence is related to the fact that water availability and SN contrast with factors related to potential water loss (ETo, SR, and Tmean). Thus, this directly affects the set of agronomic variables linked to forage canopy growth (FA, LBA, TPD, and CHI), which are important factors in herbivore production systems. These important factors contrast with DMA (Figure 5), which is the result of plant tissue death and is viewed negatively in intensive production systems (Carnevalli et al., 2006).

Multiple regression analysis is a particular case of canonical correlation that can be used to understand which agrometeorological variables are important in the composition of empirical models for estimating productive variables, such as biomass production and tiller density (Qiu et al., 2016; Mathieu and Aires, 2018). In this case, these productive variables would be more related to data on water availability, particularly ETo, WI, and SN. High temperature is an important growth factor for C4 cycle grasses that is related to the increased metabolic activity of plants; however, water stress can be a harmful factor for plant development (Prasad et al., 2011; Mathieu and Aires, 2018). In forage plants, this may be linked to increased plant material death in contrast to biomass accumulation, as observed in the canonical agronomic variable. This was mainly due to the greater variability of the data in the environmental conditions during the conduct of the tests, reflecting the lack of control of the water supply of the crop in the GG.IGA.15 and GG.IGA.17-18 tests (Figure 5). The aspects related to the management of the GG.IGA.15 study, considering regrowth periods that ranged from 14 to 49 days, caused this cultivation condition to present high variability in the canonical agronomic variable which leads to the high variability of forage canopy structural conditions (Macedo et al., 2021).

The variability of observations from these assays, both by principal component and canonical correlation analysis, is related to the response characteristics of grasses to environmental conditions. Unlike many other species, such as those of the genus *Urochloa* (syn. *Brachiaria*), grasses of the genus *Megathyrsus* are more responsive to edaphoclimatic conditions and highly productive when these conditions are favorable, so they are used in intensive production systems under grazing (Pontes et al., 2016). Discriminant function 1, which had the greatest power to separate the studies, discriminated the trials mainly by location (Figure 6). In other words, the difference between the climatic regions (humid tropical and semi-arid) promotes a greater power of distinction than the studied cultivars (Guinea and Massai grass).

It is possible to visualize the difference between experiments GG.PEN.03 and GG.PEN.05–06 (Table 3) despite having the same locations. This was likely due to climatic differences between the times the tests were conducted, mainly with regard to temperature, which was 3.52°C higher in the period from 2005 to 2006 compared to the experimental period in 2003 (Table 1). Regarding the tests in Igarapé-Açu, the temperature difference was only 0.65°C between GG.IGA.15 and GG.IGA.17-18. There was a difference between these two studies regarding DF1, which did not occur between GG.IGA.15 and MG.IGA.15 (Table 3), which that took place in the same year. Thus, the importance of climate-related variables in the composition of DF1 is evident. As both Pentecoste trials were adequately supplied with water through irrigation, the effect of temperature change may have been the main factor in discriminating these trials (Table 2). Temperature influences the acceleration of the physiological processes of the plant, resulting in greater forage accumulation, stem elongation, and dead material (Ivory and Whiteman, 1978). DF1 was more clearly observed by the greater effect of Tmean and SA, since the effect of temperature on stem elongation is well evidenced in grasses (Liu et al., 1998; Pierre et al., 2011; Yang et al., 2014).

The discrimination of the trials regarding the forage cultivar was based on the discriminant function 2. This function accounted for only 19.20% of the variation in the separation power of the studies. It is possible to observe the strong influence of agronomic variables on this discriminant function, mainly regarding the effects of TPD, FA, DMA, and LBA. Massai grass dominated for these variables because it presented higher TPD and biomass production compared to Guinea grass (Veras et al., 2020). In contrast, Guinea grass presented greater values in height increment, as this cultivar is larger than Massai grass. It is possible to see here the negative relationship between canopy height and TPD for tropical grasses observed in recent studies (Macedo et al., 2021; Xiliang et al., 2021), in which canopies with greater height tend to have lower TPD. Unlike DF1, DF2 did not differ in the Guinea grass trials in Igarapé-Açu at different times (Table 3). The highest values for the MG.FOR.09 study showed the strong production capacity of the Massai grass cultivated under conditions of high temperature and radiation in low-latitude regions (Table 1). Associated with this, the high availability of water explained the stronger positive correlation of WI with DF2 than the other variables (Figure 6).

Through a multivariate approach with experimental test data, we show that in production systems based on the cultivation of grasses of the genus *Megathyrsus* in regions located near the equator, despite the similarities in energy supply in terms of radiation and temperature, water availability, and nutrient supply are the determining factors for biomass accumulation. Therefore, these factors should be prioritized in future studies modeling forage biomass accumulation. However, in tropical pastoral ecosystems, forage plant management also had a determining effect on the accumulation of total biomass and its components, which should be considered in studies of relationships between productive variables and pasture characterization.

## Data Availability Statement

The data analyzed in this study is subject to the following licenses/restrictions: Dataset will be used in future modeling research by the team. Requests to access these datasets should be directed to anibal.cr@ufra.edu.br.

## Author Contributions

VM: conceptualization, methodology, formal analysis, investigation, data curation, and writing—original draft. NL, AC, ML, RS, and JC: investigation, resources, data curation, and writing—review and editing. CF, MC, and AR: conceptualization, resources, writing—review and editing, supervision, and project administration. All authors contributed to the article and approved the submitted version.

## Funding

This study was financed in part by the Coordenação de Aperfeiçoamento de Pessoal de Nível Superior—Brazil (CAPES; Finance Code 001).

## Conflict of Interest

The authors declare that the research was conducted in the absence of any commercial or financial relationships that could be construed as a potential conflict of interest.

## Publisher’s Note

All claims expressed in this article are solely those of the authors and do not necessarily represent those of their affiliated organizations, or those of the publisher, the editors and the reviewers. Any product that may be evaluated in this article, or claim that may be made by its manufacturer, is not guaranteed or endorsed by the publisher.
